# Genetic Abnormalities in Extramedullary Multiple Myeloma

**DOI:** 10.3390/ijms241411259

**Published:** 2023-07-09

**Authors:** Roisin McAvera, John Quinn, Philip Murphy, Siobhan Glavey

**Affiliations:** 1 Department of Pathology, Royal College of Surgeons in Ireland, D09 YD60 Dublin, Ireland; roisinmcavera@rcsi.com; 2 Department of Haematology, Beaumont Hospital, D09 V2N0 Dublin, Ireland; 3 School of Medicine, Royal College of Surgeons in Ireland, D02 YN77 Dublin, Ireland

**Keywords:** extramedullary myeloma, genomics, high-risk, multiple myeloma

## Abstract

Extramedullary multiple myeloma (or extramedullary disease, EMD) is an aggressive form of multiple myeloma (MM) that occurs when malignant plasma cells become independent of the bone marrow microenvironment. This may occur alongside MM diagnosis or in later stages of relapse and confers an extremely poor prognosis. In the era of novel agents and anti-myeloma therapies, the incidence of EMD is increasing, making this a more prevalent and challenging cohort of patients. Therefore, understanding the underlying mechanisms of bone marrow escape and EMD driver events is increasingly urgent. The role of genomics in MM has been studied extensively; however, much less is known about the genetic background of EMD. Recently there has been an increased focus on driver events for the establishment of distant EMD sites. Generally, high-risk cytogenetic abnormalities and gene signatures are associated with EMD, alongside mutations in RAS signalling pathways. More recently, changes in epigenetic regulation have also been documented, specifically the hypermethylation of DNA promoter regions. Therefore, the focus of this review is to summarize and discuss what is currently known about the genetic background of EMD in MM.

## 1. Introduction

Multiple myeloma (MM) is the second most common blood cancer worldwide and is characterized by the clonal proliferation of malignant plasma cells in the bone marrow (BM) [1,2]. These plasma cells secrete a monoclonal immunoglobulin (Ig), often known as M-protein, which can lead to organ dysfunction, anaemia, renal impairment, and bone lesions. Unfortunately, MM is incurable as eventually all patients relapse, with a median overall survival of 6 years [1,3]. MM is an extremely heterogeneous disease resulting from the accumulation of genetic aberrations that give rise to oncogenic transformation. MM is preceded by well-characterised pre-malignant-stage monoclonal gammopathy of undetermined significance (MGUS) and smouldering MM (SMM), and each have their own genetic background [4]. Progression to symptomatic MM is a result of clonal evolution, and this can further drive patients to become refractory/relapse. In rare cases, patients present with extramedullary disease (EMD), an aggressive form of MM that has become independent of the bone marrow microenvironment and may infiltrate other organ systems. EMD may occur alongside MM at diagnosis in around 7% of patients or manifest at later stages of relapse in 6–20% [5]. EMD is considered to be a high-risk factor, with reports of extremely poor prognosis of no more than 3 years in patients after autologous stem cell transplant (ASCT) and less than 1 year in refractory patients [6,7].

When discussing EMD, it is important to acknowledge that there is controversy over its precise definition. Some groups define it as only extraosseous soft tissue masses that result from haematogenous spread (known as ‘extraosseous’ EMD) [7,8]. Alternatively, a broader definition often used also includes bone-related (or paraskeletal) plasmacytomas, also known as ‘osseous’ or ‘bone-related’ EMD [7,8]. Many studies have included both but typically classify them as two different subtypes for comparisons as, generally, extraosseous EMD is associated with inferior prognosis [6,9]. Solitary plasmacytomas are explicitly excluded from EMD definition as these can occur in the absence of MM diagnosis [8]. Additionally, plasma cell leukaemia (PCL) is an aggressive form of MM that appears when the presence of clonal plasma cells in peripheral blood is greater than 20% [1]. However, it is also excluded from the definition of EMD since it is characterized as a unique entity with a defined clinco-pathological state and established treatment options [8]. EMD is most often diagnosed using sensitive imaging techniques such as magnetic resonance imaging (MRI) and positron emission tomography/computerised tomography (PET/CT) [8].

The introduction of new therapies for MM has been invaluable for improving patient outcomes. However, emerging research suggests that, in the era of novel agents, the incidence of EMD is still increasing, with an observed overall incidence rise from 6.3% of MM patients in 2005 to 23.5% of MM patients in 2014 [5]. Despite the heterogeneity of MM, we can stratify patients into various molecular risk subgroups by using cytogenetics or gene expression profiling (GEP) [1,10,11]. Conversely, understanding the genetic background and biology of EMD is only beginning, and most of the studies reported in the literature used small patient cohorts. Cytogenetic abnormalities are present, and differences have been observed between classic MM and EMD. In this review, we will summarize and discuss the current understanding of the genetic background of EMD, where possible distinguishing between the different types of EMD. A summary of the most relevant results provided by the main genomic studies carried out in extramedullary myeloma is presented in Table 1.

## 2. Cytogenetic Abnormalities

Cytogenetic abnormalities are a hallmark of MM, with 90% of patients presenting with such aberrations at diagnosis [27]. These occur due to chromosomal instability and can both initiate disease and establish clonal evolution seeding with respect to bone marrow and, eventually, EMD sites [28]. The initiation of cytogenetic abnormalities is most commonly attributed to trisomies of odd-numbered chromosomes (hyperdiploidy) and translocations involving the IGH gene locus on chromosome 14q32. Secondary cytogenetic events are more prevalent in later disease stages, and common examples include del(13), del(17p13), gain(1q), and del(1p). These abnormalities can be detected using fluorescence in situ hybridization (FISH) and may be used to guide patient prognosis. For example, the Revised-International Staging System (R-ISS) incorporates the presence of high-risk abnormalities, such as t(4;14), t(14;16), or del(17p), to stratify patients into three prognostic groups [29]. The 5-year survival rates for R-ISS stages I, II, and III are 82%, 62%, and 42%, respectively, highlighting the differential disease severity and prognoses for each patient [29]. Additionally, the Mayo Stratification for Myeloma and Risk-adapted Therapy (mSMART) guidelines use several more genetic factors to guide genetic risk (Table 2) [30]. The overall survival for high-risk MM patients is generally less than 3 years, whilst standard-risk patients exhibit survival rates of 7–10 years [1]. Given the importance of cytogenetic events in MM pathogenesis, most studies on EMD have aimed to establish their incidence in this setting.

Generally, most studies have shown increasing cytogenetic complexity at EMD sites compared to BM, demonstrating that EMD is an aggressive form of MM with defined clonal evolutionary properties [16,24]. Specifically, many of these additional abnormalities are high-risk, such as t(4;14), del(17p13), del(13) and chromosome 1 aberrations, in keeping with the concept that high-risk cytogenetic features drive relapse not only in the bone marrow but also at selective EMD sites. Besse at al. [16] used FISH to detect abnormalities in paired samples from BM plasma cells and EMD sites for 12 patients. In these cases, del(13q14) and 14q32 disruptions were more prevalent in BM sites compared to EMD, but the frequency of genomic events was increased in patients at the time of EMD diagnosis compared to samples collected previously. Moreover, in paired samples, gain(1q) occurred in both BM and EMD plasma cells in 66.7% of all cases. However, when comparing unrelated BM samples to the overall EMD samples, an increased frequency of t(4;14) was observed in EMD [16]. Alternatively, a retrospective study showed no difference in the prevalence of del(13q14) and t(4;14) in diagnostic BM aspirates collected from patients with or without EMD; however, plasma cells from EMD sites were not assessed [15]. In this study, there was a higher frequency of del(17p13) and amp(1q21) in EMD vs. non-EMD (31% vs. 13% and 55% vs. 31%, respectively). The authors also reported cytogenetics for patients who present with EMD at diagnosis (21 patients) vs. at relapse (8 patients), and there were no significant differences between the two [15]. Another retrospective study described that the presence of any IGH translocation, del(13/13q), and del(17) in BM at diagnosis did not predict progression to EMD (neither osseous nor extraosseous) in a cohort of 117 patients treated with bortezomib with or without lenalidomide [31]. However, the authors did note that no patients with t(11;14) developed extraosseous EMD, and this was also reported in an earlier study [32]. This corroborates the consensus that t(11;14) is generally associated with standard-risk disease. Overall, studies such as these demonstrate no unitive underlying genomic process accounting for the diverse and unpredictable nature of EMD, likely indicating that deeper and broader genomic studies are required to truly profile this form of MM.

Kriegova et al. [23] performed whole-genome optical mapping on BM plasma cells in a small cohort of 11 newly diagnosed MM patients, 4 of which presented with bone-related EMD. This method enabled the detection of large chromosomal rearrangements as well as small structural variants and copy number variations. Strikingly, chromosome 1 abnormalities were present in all EMD patients and consisted of various intrachromosomal rearrangements that resulted in copy number changes of genes, including recurrent regions encompassed by del(1p32) and gain(1q21). Additionally, del(17p13) was detected in two of the EMD patients but not in any patients presenting without EMD, which is indicative of a trend similar to that of other studies linking del(17p13) to EMD [12,23]. Promisingly, where optical mapping revealed changes in common MM-associated regions, the FISH results were able to confirm these findings in the majority of cases. Most of the studies discussed did not explore del(1p32) despite it largely being considered a high-risk MM abnormality [33,34]. Nevertheless, another study using a small cohort of EMD patients revealed that del(1p32) and gain(1q21) were common occurrences both in BM plasma cells and at EMD lesion sites, suggesting that chromosome 1 abnormalities may indeed be an important factor [20]. Moreover, gain(1q) was associated with poor survival in EMD patients, with the number of extra copies being proportional to worsened survival rates [23,24]. Together these findings suggest chromosome 1 abnormalities may play a role in the initiation and progression of high-risk EMD.

Chromothripsis is a catastrophic event involving a maximum of two chromosomes whereby chromosomes are shattered and rejoined at random, resulting in tens to hundreds of chromosomal rearrangements [35]. A recent WGS study revealed that 20–30% of newly diagnosed MM patients have chromothripsis (higher than previously thought), and this is an adverse prognostic marker [36,37]. Chromothripsis has also been proposed as a prognostic marker in EMD; however, currently, it has only been described in one patient [19]. This patient presented with chromothripsis of chromosome 18 in BM plasma cells at diagnosis. This consisted of six breakpoints including several deletions and amplifications, with five to six copies of 18q21 detected [19]. 18q21 harbours many important genes associated with haematological malignancies, including anti-apoptotic protein BCL-2. As more in-depth genomic analyses are performed for EMD, more complex chromosomal structural variations may be identified.

## 3. Altered Gene Expression

In the last decade, genomic risk-stratification in MM has started to incorporate gene expression profiling (GEP) more routinely. GEP enables the identification of molecular subgroups based on specific gene signatures, and our group has previously discussed some of these in greater detail [10]. Although there are numerous gene signatures that have been described, the two most commonly employed for MM risk stratification are the UAMS-70/GEP-70 and SKY92 signatures [34]. Both signatures accurately identify around 10–15% of MM patients as high-risk with poor predicted prognosis, and the detection of SKY92 using Affymetrix gene chip has been validated as an in vitro diagnostic test [10,34,38,39,40]. Additionally, patients classified as high-risk by these GEP tools are more likely to present with high-risk cytogenetics [34]. There are very limited studies published on the gene expression profile of EMD, but like with cytogenetic profile, it seems that MM patients classified as high-risk by GEP are more likely to experience EMD.

A retrospective analysis of almost 2000 MM patients compared the baseline clinical and molecular characteristics of patients who presented with EMD, either at diagnosis or relapse, with patients without EMD [41]. It is not clear exactly what definition of EMD was used. However, the risk subgroups were determined using GEP-70 and GEP-80 signatures and revealed an association between EMD and high-risk disease [41]. In GEP high-risk patients who received a transplant, 10.8% showed EMD within 5 years compared to only 2% in standard-risk [41]. It is important to note that most of the 70 genes making up GEP-70 are located on chromosome 1, with most upregulated and downregulated genes being on 1q and 1p, respectively [38]. Therefore, given the occurrence of gain(1q) and del(1p) in EMD patients, it is not surprising that GEP-70 may predict EMD [20,24]. Additionally, this study also used a gene expression-based centrosome index, which was first described by Chng et al. [42]. Briefly, this uses the expression of four centrosome genes—CETN2, TUBG1, PCNT1, and PCNT2—to predict centrosome amplification and poor prognosis [42,43]. A high centrosome index was associated with the presence of EMD at diagnosis [41].

Sevcikova et al. [14] investigated the expression of a small subset of 15 genes included in the GEP-70 signature in clinically high-risk MM patients who relapsed within 24 months of diagnosis compared to patients with EMD. Notably, four genes—CKS1B, CTBS, NADK, and YWHAZ—were significantly altered in BM plasma cells in EMD patients compared to MM [14]. Moreover, authors described changes in 9/15 genes when comparing EMD tumour cells with paired BM plasma cells, signifying spatial heterogeneity within patients [14]. Despite identifying substantial differences in gene expression, no validation studies were performed to determine their use as biomarkers for EMD. Additionally, it was not explained why only these specific 15 genes were selected for investigation. In a recent study, a microarray assay for long non-coding RNAs (lncRNAs) identified NEAT1 as highly expressed in EMD plasma cells and showed that it was associated with gain(1q21) [25]. NEAT1 can regulate gene transcription in key processes, including DNA repair and the cell cycle, and in this study, NEAT1 knockdown led to a reduction in the proliferation of in vitro myeloma cell lines [25].

Ryu et al. [22] performed an in-depth genomic study combining exon sequencing, RNA-sequencing, and single-cell RNA sequencing (scRNA-seq) to characterise extramedullary progression. The group used single-cell approaches to elegantly decipher different immune cell populations in MM; however, for the purposes of this review, this will not be discussed. Interestingly, alterations in the transcription of several pathways were observed upon extramedullary progression, and these included genes involved in the cell cycle, glycolysis, oxidative phosphorylation, proteasome, and antigen presentation [22]. The transcriptome of BM cells at relapse/refractory stage were more similar to the EMD samples than diagnostic BM, suggesting that, in these cases, aggressive transformation occurred within the BM initially [22]. Additionally, the transcriptional expression of IL-6 and the IL-6 receptor was greater in EMD cells and refractory BM cells, suggesting that IL-6 signalling plays a role [22]. Similarly, Sun et al. [26] very recently used scRNA-seq to characterize the transcriptome of various cell populations responsible for progression and metastasis in EMD. This group focused on three patients with a specific form of extraosseous EMD known as myelomatous effusion (ME), which manifests as plasma cells in bodily fluids such as pleural effusion and ascites. Likewise, in their study, differences in immune cell populations were revealed throughout BM, peripheral blood (PB), and EMD sites [26]. Differentially expressed gene pathways enriched in EMD samples included oxidative stress, metabolic stress, protein kinase regulator activity, and protein folding responses. From these analyses, leukocyte immunoglobulin-like receptor subfamily B4 (LILRB4) was identified as upregulated in extramedullary cells and could be a potential biomarker for such disease progression [26]. As more transcriptomic data are gathered for EMD in MM, we may move towards better methods to understand this phenotype and enhance the potential to stratify patients at risk.

## 4. Mutational Landscape

MM patients also present with recurrent somatic mutations, most commonly RAS pathway mutations such as NRAS, KRAS, and BRAF, which occur in around 40% of newly diagnosed MM and typically result in activation of MAPK signalling [44]. TP53 mutations are observed in a very small subset of newly diagnosed patients; however, incidence at relapse is increased [45]. Additionally, studies have identified mutations in other genes such as ATR, ATM, PTPN11, TRAF3, and IDH1/2 genes [44,46]. It is well known that progression from pre-malignant MGUS through to symptomatic MM is a result of a clonal evolution whereby the mutational landscape is shaped by the fitness of various subclones [4]. For example, RAS mutations are often present at extremely subclonal levels at the SMM; however, clonality is increased at the MM and relapse stages [4]. Additionally, whole exome sequencing (WES) studies in paired MM samples from before and after treatment revealed a change in clonal composition in 82% of patients, with increased incidence of RAS and TP53 mutations being most common [28]. Given the impact of treatment pressure on clonal evolution, it is possible that this is also contributing to the rising incidence of EMD.

In a patient presenting with multi-drug refractoriness and EMD relapse, whole genome sequencing (WGS) was performed on the EMD neck mass in an attempt to find novel therapeutic targets [13]. This revealed extreme genomic instability, including 271 nonsynonymous, somatic point mutations, including in KRAS and ATM and, for the first time, CRBN, which has since been linked to drug resistance in many studies (usually resistance to immunomodulatory agents) [47,48]. This reinforces the theory that EMD is extremely high-risk and unresponsive to therapy.

De Haart et al. [49] performed next-generation sequencing (NGS) using a panel targeting 50 known oncogenes and tumour suppressor genes on 14 relapsed EMD patients. Where possible, the authors compared tumour DNA from formalin-fixed, paraffin-embedded (FFPE) diagnostic BM samples to relapse BM and soft tissue EMD samples. RAS mutations were reported in 69% of the EMD samples, and in the majority of cases, an identical mutation was already present at diagnosis, perhaps suggesting that it could be predictive of eventual EMD [49]. Similarly, in another study, a female with aggressive EMD relapse presented at diagnosis with a missense NRAS mutation [19]. Since then, several other studies utilising targeted DNA sequencing have also reaffirmed the high prevalence of RAS mutations in patients with EMD [20,22].

Overall, de Haart et al. [49] reported that TP53 mutations were associated with relapsed samples from either extramedullary sites or BM; however, it should be noted that for several of these patients, NGS could not be performed at diagnosis, thus potentially skewing this result. Moreover, other studies have detected TP53 mutations at diagnosis in patients who go on to relapse extramedullary [19]. One patient did not present with typical RAS or TP53 mutations but rather mutations in APC and ATM, which were detected in BM at diagnosis and in their EMD lesion. Another patient presented with only a KIT mutation at diagnosis, which remained present at relapse, alongside a KRAS mutation only in EMD cells. As mentioned, ATM mutations have been observed in MM before and can lead to deregulated DNA repair and genomic instability [50,51]. APC and KIT mutations were not previously described in MM, and the authors do point out that, since healthy patient tissue was not analysed, these may not be associated with pathogenesis [49].

Given the invasive nature of BM biopsies, an attractive alternative is the use of liquid biopsies for mutational analysis. Long et al. [21] performed a small study on eight EMD patients using paired samples from EMD tissue, BM, and circulating tumour DNA (ctDNA) from plasma. Again, RAS and TP53 mutations were most commonly detected, but interestingly, mutations found in EMD tissue were more concordant with those found in ctDNA than in BM. This approach had been used previously by Mithraprabhu et al. [17] to monitor and track disease progression in a patient with EMD, revealing significant spatial heterogeneity and clonal evolution over a two-year period. Moreover, ctDNA levels in blood were correlated with tumour burden [17]. Together, these findings suggest that this non-invasive approach is adequate for the genetic characterisation of EMD and could be used increasingly in the future, especially in cases where the EMD tumour is not easily accessible.

## 5. Epigenetic Changes

Gene transcription is tightly controlled by epigenetic regulation (usually DNA methylation and histone modifications) to either transcriptionally silence or express genes. Therefore, in addition to gene-level alterations, epigenetic dysregulation can also contribute to tumour heterogeneity and pathogenesis. Compared to gene expression and cytogenetics, even less is known about the epigenome in extramedullary myeloma. However, what we do know is that up to 53% of MM patients present with mutations in genes involved in epigenetic regulation, and these have prognostic significance [52]. Moreover, Walker et al. showed that differential CpG methylation patterns are correlated with the transition from MGUS to MM and, likewise, to PCL [53]. Therefore, it is likely that epigenetic alterations may also contribute to the presentation of EMD.

To date, only one study has directly investigated the epigenome of MM patients with EMD. Yao et al. [18] presented a small study involving two MM patients who presented without EMD but later relapsed both in BM and with bone-related EMD. The methylation profiles of five disease-related genes (SHP1, CDK2NA, CDH1, CD56, and CXCR4) were determined in diagnostic BM plasma cells, as well as multiple relapse samples from both BM and EMD sites. Interestingly, in diagnostic BM, there was no evidence of methylation in any of the five genes; however, in the relapse samples this had changed. Notably, SHP1 methylation was detected in both patients in EMD cells at relapse, suggesting it may be a common factor in EMD progression. SHP1 hypermethylation has previously been described in MM and can lead to the constitutive activation of JAK/STAT3 signalling and disease progression [54,55]. Therefore, it is likely that a similar mechanism may also contribute to BM escape and EMD progression. Additionally, in one patient, CDKN2A and CDH1 methylation was also present at relapse [18]. Importantly, the authors note that the methylation profiles differed between sites at relapse, with BM at relapse showing varying gene methylation compared to relapse EMD in both patients, indicating epigenetic spatial heterogeneity. Through Sanger sequencing of the unique CDR3 region of the IGH gene of the diagnostic MM clone and EMD clone, the authors showed that the EMD had evolved from the original MM, thus demonstrating a case of clonal evolution.

More recently, an interesting study by Farre et al. [56] developed a novel model to study EMD. This study involved a patient-derived orthotopic xenograft (PDOX) from cutaneous EMD plasma cells, which was used to study various aspects of EMD, including genetics, epigenetics, and drug response. In addition to a highly disturbed genome, whole genome methylome analysis revealed significant changes in CpG methylation in the EMD PDOX model when compared to both normal plasma cells and malignant MM cells from a published dataset. Consistent with Yao et al. [18], hypermethylation was associated with EMD, with 89.6% of CpGs showing hypermethylation in EMD PDOX compared to the MM dataset. However, it should be noted that the PDOX model was only representative of one patient and, ideally, in the future, the use of larger cohorts would be beneficial. Additionally, the PDOX model was compared to unrelated published samples rather than healthy or MM cells from the patient it was derived from. Including this comparison would strengthen the research findings. Additionally, comparing the PDOX methylome to that of the isolated EMD cells alone would also account for any changes induced by the model itself.

Ultimately, there is still much to learn about how the epigenome may contribute to EMD, and future studies should aim to determine whole epigenome status to identify novel targets. Of the limited studies published in this area, both have focused only on DNA methylation status since it is the most well-described epigenetic marker. However, histone methylation and acetylation are also important markers that should be studied in the future. Moreover, the use of histone deacetylase (HDAC) inhibitors for the treatment of relapsed/refractory MM shows that epigenetic regulation can be a therapeutic target [57].

## 6. Conclusions and Future Directions

**Figure 1:**
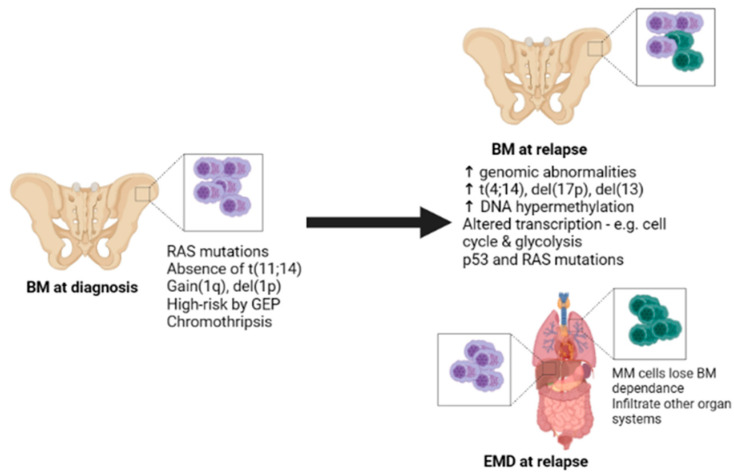
Current understanding of genetic abnormalities in extramedullary myeloma. Summary of main genetic abnormalities described in EMD. Patients who relapse with EMD may present at diagnosis with high-risk cytogenetic abnormalities, RAS mutations, high-risk gene signatures, and potentially chromothripsis. Generally, upon relapse, the frequency of genomic abnormalities is increased as clonal selection occurs and is higher in EMD lesions compared to BM. More high-risk factors are present, and increased DNA hypermethylation may contribute to EMD. Spatial heterogeneity may also occur, both between BM and EMD and various EMD lesions.

EMD in MM is an extremely high-risk factor with prognostic significance, and its incidence is on the rise. The genomic and epigenomic background of EMD in MM is not well-characterised, and the few studies performed to date have been relatively small in scale. High-risk cytogenetic events and gene signatures are associated with EMD, with a high prevalence of t(4;14), del(13) and del(17) and chromosome 1 abnormalities being reported (Figure 1). However, precisely how these influence EMD pathogenesis is unknown. Moreover, there is a lack of consensus regarding which cytogenetic abnormalities are assessed between clinical sites; thus, it is hard to directly compare results from different studies. Likewise, different gene signatures may also be used for risk classification across studies. Early studies indicate alterations in epigenetic regulation, specifically DNA hypermethylation. In the future, global epigenome status may reveal more specific information about how the epigenome is altered in EMD and perhaps pave the way for use of demethylating agents in this setting. Similarly, regarding somatic mutations, it is hard to compare studies as most have opted for targeted panel approaches. In-depth genome-wide, comprehensive, and combinational genetic sequencing approaches like those used by Ryu et al. [22] and Smetana et al. [19] are necessary to really understand EMD; however, such studies come at a large expense and are often not feasible, particularly for larger cohorts. As an alternative, liquid biopsy methods may help us to understand where BM and/or EMD biopsies are too invasive [17,21].

From reviewing the existing literature, we observed that a major discrepancy in most studies is that genetic sequencing was performed only on BM plasma cells, mostly at diagnosis but sometimes upon relapse. However, many studies lacked paired EMD samples. Future studies should aim to perform genetic testing on cells from individual EMD lesions as this will give the best insight into disease progression and clonal evolution, particularly since some studies have reported spatial heterogeneity [17,18,58]. Moreover, if patients present with multiple relapses, ideally, BM and EMD lesions would be sequenced each time. There is also a scarcity of published studies comparing EMD at diagnosis compared to those for EMD presenting at relapse; investigating this may also reveal differences in pathogenesis. Given the evidence for spatial and temporal heterogeneity in EMD progression, we propose that future work should aim to characterize the influence of novel therapies on malignant clonal selection in the context of EMD.

Ultimately, research in the field of MM has been conducted with the aim of identifying personalised, risk-adapted strategies; however, these are extremely lacking in MM compared to other cancers. It is beyond the scope of this review to discuss treatment approaches in depth; however, ultimately, there are no therapies specifically aimed at the treatment of EMD. Moreover, little is known about how current therapies may influence the development of EMD and its genetic and epigenetic composition. Hopefully, as more genetic alterations are uncovered, some of these may translate into disease biomarkers and therapeutic targets.

## Figures and Tables

**Table 1:** Summary of the most relevant genomic studies performed to characterize extramedullary myeloma.

Study/Reference	Patient Cohort Description	Sample Type(s)	Methodologies	Results Summary
Billecke et al., 2013 [12]	36 MM patients, 17 with EMD at diagnosis or relapse; 11 bone-related and 6 extraosseous	BM	FISH	High incidence of del(17p) in both EMD groups compared to non-EMD.
Egan et al., 2013 [13]	One relapsed, refractory patient with extraosseous EMD	EMD	WES, WGS, RNA-seq	Highly altered genome revealed. Of note were mutations in ATM, KRAS, NFKB2, and PSMG2. First report of CRBN mutation in EMD and low CRBN gene expression.
Sevickova et al., 2015 [14]	18 patients, 9 with ‘high-risk’ * MM and 9 with EMD	‘High-risk’ patients; BM EMD patients; paired BM and EMD cells	Quantitative PCR	Four GEP-70 genes deregulated in EMD BM vs. high-risk MM BM samples. Within patients with EMD, nine genes were deregulated in EMD tissue compared to BM.
Qu et al., 2015 [15]	Retrospective study of 300 patients, 41 of which had EMD at diagnosis or progression	BM	FISH	Del(17p13) and amp(1q21) associated with EMD.
Besse et al., 2016 [16]	31 EMD patients either at MM diagnosis or relapse, 15 bone-related, 16 extraosseous	Paired BM and EMD	FISH	In unrelated samples, higher incidence of t(4;14) in EMD compared to BM. In paired samples, gain(1q) was frequent in BM and EMD.
Mithraprabhu et al., 2018 [17]	One patient with relapse extraossesous EMD	Paired EMD and PB (several time-points)	WES	ctDNA can be used to track EMD progression and clonal evolution. Both spatial and temporal heterogeneity observed. NRAS mutation observed at clonal and subclonal levels.
Yao et al., 2018 [18]	Two MM patients with relapse EMD	Diagnostic BM, paired relapse (BM and EMD), and relapse PB for one patient	Methylation-specific PCR	SHP1 methylation detected in both patients only at relapse. Evidence of spatial methylation heterogeneity in MM/EMD.
Smetana et al., 2018 [19]	One MM patient with EMD at relapse	BM (diagnostic)	Array-CGH, Targeted NGS	Patient presented with huge chromothripsis of chromosome 18 and mutations in NRAS, RAF1, TP53, CUX1 and POU4F1 before progression to EMD.
Liu et al., 2020 [20]	10 patients with EMD, 4 at diagnosis and 6 at relapse	BM & EMD, paired where possible	FISH, Targeted NGS, SNP microarray	Gain(1q21) and del(1p32) common in BM and EMD lesions. High prevalence of RAS mutations
Long et al., 2020 [21]	10 MM patients without EMD, 8 MM patients with EMD	Paired BM, EMD & plasma	Targeted NGS	ctDNA may be used for mutational characterization of EMD. Evidence of spatial heterogeneity between BM and EMD/ctDNA.
Ryu et al., 2020 [22]	15 MM patients, 5 with EMD	BM and EMD, paired samples for three patients	scRNA-seq, WES	RAS pathway mutations common in BM and EMD samples. Transcriptional alterations observed in the cell cycle, glycolysis, oxidative phosphorylation, proteasome, and antigen presentation upon EMD progression. Upregulated IL-6 signalling in EMD.
Kriegova et al., 2021 [23]	11 newly diagnosed MM patients, 4 with bone-related EMD	BM	Whole-genome optical mapping	Large intrachromosomal rearrangements within chromosome 1 detected in all EMD patients.
Xia et al., 2022 [24]	30 patients with EMD; 19 bone-related and 11 extraosseous	Paired BM and EMD	FISH	Higher frequency of genomic aberrations in EMD tissue vs. BM. Higher prevalence of gain(1q) and P53 deletion in EMD, and higher in bon-related EMD compared to extraosseous.
Chen et al., 2023 [25]	3 relapsed MM patients with bone-related EMD	Paired BM (diagnostic) and EMD	RNA-seq, scRNA-seq, microarray	Identified lncRNA NEAT1 as upregulated in EMD and confirmed its association with aggressive disease in vitro.
Sun et al., 2023 [26]	3 patients with EMD at progression (specifically ME)	Matched BM, PB, and EMD	scRNA-seq	Determined transcriptome changes associated with EMD progression, specifically plasma cell proliferation and migration. Identified LILRB4 upregulation in ME compared to BM and confirmed effects in vitro.

* High-risk defined as patients with relapse within 24 months of diagnosis. Abbreviations: MM—multiple myeloma; BM—bone marrow; EMD—extramedullary disease; FISH—fluorescence in situ hybridization; lncRNA—long non-coding RNA; ME—myelomatous effusion; NGS—next-generation sequencing; PB—peripheral blood; PCR—polymerase chain reaction; scRNA-seq—single-cell RNA sequencing; SNP—single-nucleotide polymorphism WES—whole exome sequencing; WGS—whole genome sequencing.

**Table 2:** mSMART 3.0 classification of newly diagnosed Multiple Myeloma (MM).

Risk Classification	Criteria
Standard-Risk	Trisomies t(11;14) t(6;14)
High-Risk	High-risk genetic abnormalities: t(4;14) t(14;16) t(14;20) del(17p) p53 mutation gain(1q)
	R-ISS Stage III High plasma cell S-phase GEP: High risk signatures
	Double-hit MM: Any two high-risk factors Triple-hit MM: Any three high-risk factors

R-ISS—Revised International Staging System; GEP—gene expression profiling.

## Data Availability

Not applicable.
